# Supplementary material for: The COX-2/PGE2 Response Pathway Upregulates Radioresistance in A549 Human Lung Cancer Cells through Radiation-Induced Bystander Signaling
Source: Biology (Basel). 2023 Oct 25;12(11):1368. doi: 10.3390/biology12111368 (PMC10669009; doi:10.3390/biology12111368)
Supplement: Supplementary file 1 [file biology-12-01368-s001.zip › biology-2619464-supplementary-revised.pdf]

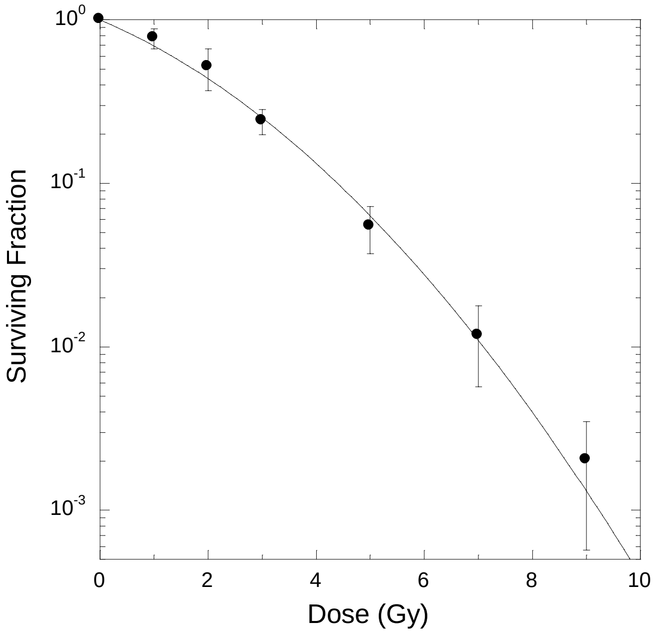

Figure S1. A549 cells were exposed to X-ray with dose ranging from 1 to 9 Gy, and the cell survival was determined by colony formation assay. The cells were prepared in T12.5 bottle flasks with 10^6 cells. Survival fractions for each dose were averaged from 7 repeated experiments. Error bars represents the standard errors. Solid line is the fitted regression line of linear Quadratic (LQ) model, S= exp (-α×D-β×D2), where S is the surviving fraction, D is the dose in Gy, and α (Gy^{-1}) and β (Gy^{-2}) are the fitting parameters. Using the α, β parameters 10% surviving dose (D_{10}) and 1% surviving dose (D_1) were calculated. Parameters α, β and D_{10}, D_1 are listed in the Table S1.

Table S1. The LQ model was employed to calculate fitting parameters: α, β and D_{10}, D_1 based on the survival rate curve present at Figure S1.

Dose (Gy)	α	β	D_{10}	D_1
X-ray	0.328±0.055	0.044±0.034	4.42±0.35	7.18±0.67
